# Comparison of Choroidal Thickness in Affected and Fellow Eyes of Treatment-Naive Retinal Vein Occlusion Patients

**DOI:** 10.7759/cureus.85119

**Published:** 2025-05-31

**Authors:** Arnab Garai, Ramanuj Samanta, Sanjeev K Mittal, Ranjeeta Kumari, Sreeram Jayaraj, Neeti Gupta

**Affiliations:** 1 Ophthalmology, All India Institute of Medical Sciences, Rishikesh, Rishikesh, IND; 2 Community and Family Medicine, All India Institute of Medical Sciences, Rishikesh, Rishikesh, IND; 3 Ophthalmology, Shri Guru Ram Rai Institute of Medical and Health Sciences, Dehradun, IND

**Keywords:** branch retinal vein occlusion, central retinal vein occlusion (crvo), choroidal thickness, enhanced depth imaging optical coherence tomography, retinal venous occlusion, spectral domain optical coherent tomography

## Abstract

Purpose: The primary purpose of the study was to compare the baseline choroidal thickness (CT) between affected eyes and fellow eyes of treatment-naive retinal vein occlusion (RVO) patients by spectral domain optical coherence tomography (SD-OCT). The secondary purpose of the study was to find any association between presenting visual acuity and CT in the affected eyes of treatment-naive RVO patients.

Methods: This was a comparative, cross-sectional study. A total of 68 eyes of 34 treatment-naive RVO patients in the age group of 40-60 years were prospectively included in the study. Individuals with refractive error beyond ±6 D and posterior segment pathology other than RVO were excluded. The baseline axial length and intraocular pressure of the affected and fellow eyes were taken into consideration so that these parameters do not have any effect on the CT. The CT at the subfoveal region, at a distance of 500 µm nasal and 500 µm temporal to the fovea, was measured on SD-OCT with enhanced depth imaging (EDI) mode.

Results: The CT at all locations was significantly higher in the affected eyes of RVO patients (sub-foveal, 500 µm nasal, 500 µm temporal, and mean CT: 326.1 ± 80.0 µm, 325.4 ± 99.7 µm, 328.1 ± 100.8 µm, and 326.5 ± 87.8 µm, respectively) compared to the fellow eyes (sub-foveal, 500 µm nasal, 500 µm temporal, and mean CT: 264.2 ± 69.2 µm, 263.1 ± 69.5 µm, 255.2 ± 72.6 µm, and 260.8 ± 68.5 µm, respectively). The presenting visual acuity (in LogMAR) did not show a statistically significant correlation with CT (p = 0.404).

Conclusion: The study demonstrated that although CT was increased in RVO patients, it did not have any significant association with presenting visual acuity in the affected eyes of RVO patients.

## Introduction

After diabetic retinopathy, retinal vein occlusion (RVO) is one of the most frequent retinal vascular disorders [1]. According to the site of the blockage, it can be further divided into central retinal vein occlusion (CRVO) and branch retinal vein occlusion (BRVO), which are both common causes of visual impairment in elderly individuals around the world [2-3]. With frequencies of 1.8% and 0.5%, respectively, BRVO is the most prevalent vascular occlusive disease and is four times more prevalent than CRVO [4].

RVO results from the development of venous thrombus, which impairs venous drainage, makes the main retinal veins tortuous, and increases the pressure within the retinal capillaries. These modifications cause the exudation of blood, fluid, and fat into the retina, which causes macular edema (ME) [5]. ME is one of the most common complications in RVO patients and can seriously impair central vision. ME has been estimated to affect around 5-15% of patients within a year after having BRVO [6].

RVO is associated with elevated vascular endothelial growth factor (VEGF) expression in microvascular endothelial cells, pericytes, and retinal pigment epithelium, particularly in the early stages of ischemia before significant pericyte loss occurs. VEGF produces vascular dilatation and increased ocular blood flow by a nitric oxide-mediated mechanism, which may also influence choroidal thickness (CT), as demonstrated in in vitro endothelial cell studies and in vivo animal models [7]. Age, gender, ethnicity, axial length (AL), refractive errors, and diurnal variation in healthy people all have an impact on CT [8-9].

Choroids can be evaluated in vivo by fundus fluorescence angiography (FFA), indocyanine green angiography (ICGA), ultrasonography, magnetic resonance imaging (MRI), optical coherence tomography (OCT), and newer tools like optical coherence tomography angiography (OCTA) [10]. The shortcomings of most of these investigation tools in measuring CT have been overcome by the easily available, non-invasive, non-contact, rapid device OCT. Other than the individual retinal layers, the fine details of the choroidal anatomy can be assessed with the advent of novel deep-penetration OCT technologies like enhanced depth imaging (EDI) mode of spectral domain (SD)-OCT or the newer swept source (SS)-OCT [11]. It can be repeated as many times as required and also provides quantitative as well as qualitative estimation of choroidal morphology.

Measurement of CT as a biomarker has been shown to be valuable in various systemic disorders, such as hypertension and pre-eclampsia, as well as in ocular diseases, such as pachychoroid disorders, myopia, and age-related macular degeneration (ARMD) [12-16]. Very few studies in the literature have documented CT in RVO patients, most of them being retrospective. Keeping the above lacunae in mind, a cross-sectional study with prospective recruitment of study participants was designed to compare the baseline CT between the affected and fellow eyes of RVO patients.

This study aims to compare baseline CT between affected and fellow eyes of treatment-naive RVO patients using spectral domain optical coherence tomography (SD-OCT), assess the association between CT and presenting visual acuity in RVO-affected eyes, and explore the clinical implications of CT alterations in RVO with implications for future research.

## Materials and methods

This hospital-based, cross-sectional study was conducted in the Department of Ophthalmology of All India Institute of Medical Sciences, Rishikesh, a tertiary care institute in North India, for 18 months, from October 2020 to April 2022. The study followed the tenets of the Declaration of Helsinki and was approved by the Institutional Ethics Committee, All India Institute of Medical Sciences, Rishikesh (approval number: AIIMS/IEC/20/638). The study group comprised patients who were diagnosed with treatment-naive unilateral RVO at the ophthalmology outpatient department of our institute during the study period.

All treatment-naive BRVO/CRVO patients aged between 40 and 60 years presenting within two months of ocular symptom onset were included in our study. Patients with bilateral RVO; best-corrected visual acuity worse than 20/400; any other co-existing ocular diseases known to affect CT; known cases of glaucoma, ocular hypertension, or neurodegenerative disorders (Parkinson’s disease, Alzheimer’s disease); those with refractive error beyond the range of ±6 D; those who had received prior anti-VEGF injections, laser, or any other intravitreal treatments; or those with a past history of intraocular surgery (except cataract surgery performed more than six months prior) were excluded from our study. Any media opacity or poor media clarity precluding good OCT images (OCT signal strength worse than 7/10) or patients with indeterminate sclero-choroidal interface on OCT were also excluded from our study.

Demographics of all the patients, including age and gender, were noted. Detailed ophthalmic history regarding the onset and duration of ocular symptoms was obtained from each patient. Coexistent systemic disease status (diabetes mellitus and/or hypertension) was noted with duration and any medication history.

All the patients underwent detailed ophthalmic evaluation, including best corrected visual acuity (BCVA) using Snellen’s chart, refraction, measurement of intraocular pressure (IOP) by Goldmann applanation tonometer (GAT), AL measurement by optical biometry (Lenstar LS 900, Haag-Streit, USA), anterior segment evaluation by slit lamp biomicroscopy, and fundus evaluation by +78 D/90 D lens as well as indirect ophthalmoscopy. A gonioscopy was performed as and when necessary.

CT was measured by the EDI mode of SD-OCT (CirrusTM, Carl Zeiss Meditec, Dublin, CA, USA) in both the affected and fellow eye of all the patients. All images were taken by a single operator well-versed in the technique. Adequate signal strength (at least 7) in each of the scanned images was ensured. The macular line raster scan protocol with EDI mode was followed to measure the CT in the affected as well as the fellow eye. The CT was measured at three locations (subfoveal, 500 µm nasal, and 500 µm temporal to the fovea) as the perpendicular distance between the hyper-reflective outer border of the retinal pigment epithelial-Bruch’s membrane layer (RPE-BM) and the sclero-choroidal interface by two experienced observers, masked to the clinical status of the eyes (affected vs. fellow), and an average of these two values was taken for consideration for analysis. The SD-OCT measurements were performed between 12:00 and 15:00 in all patients to avoid any diurnal variation of CT.

**Figure 1:**
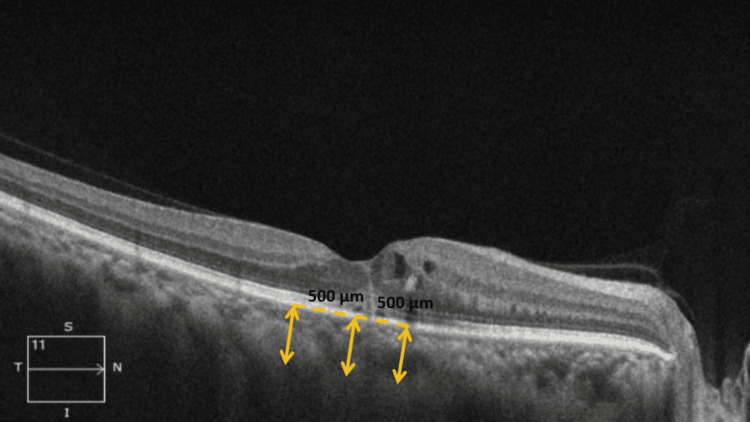
Representative SD-OCT image showing measurement of various choroidal thickness parameters of the affected eye in one of the RVO patients using EDI mode SD-OCT: spectral-domain optical coherence tomography; CT: choroidal thickness; RVO: retinal vascular occlusion; EDI: enhanced depth imaging

**Figure 2:**
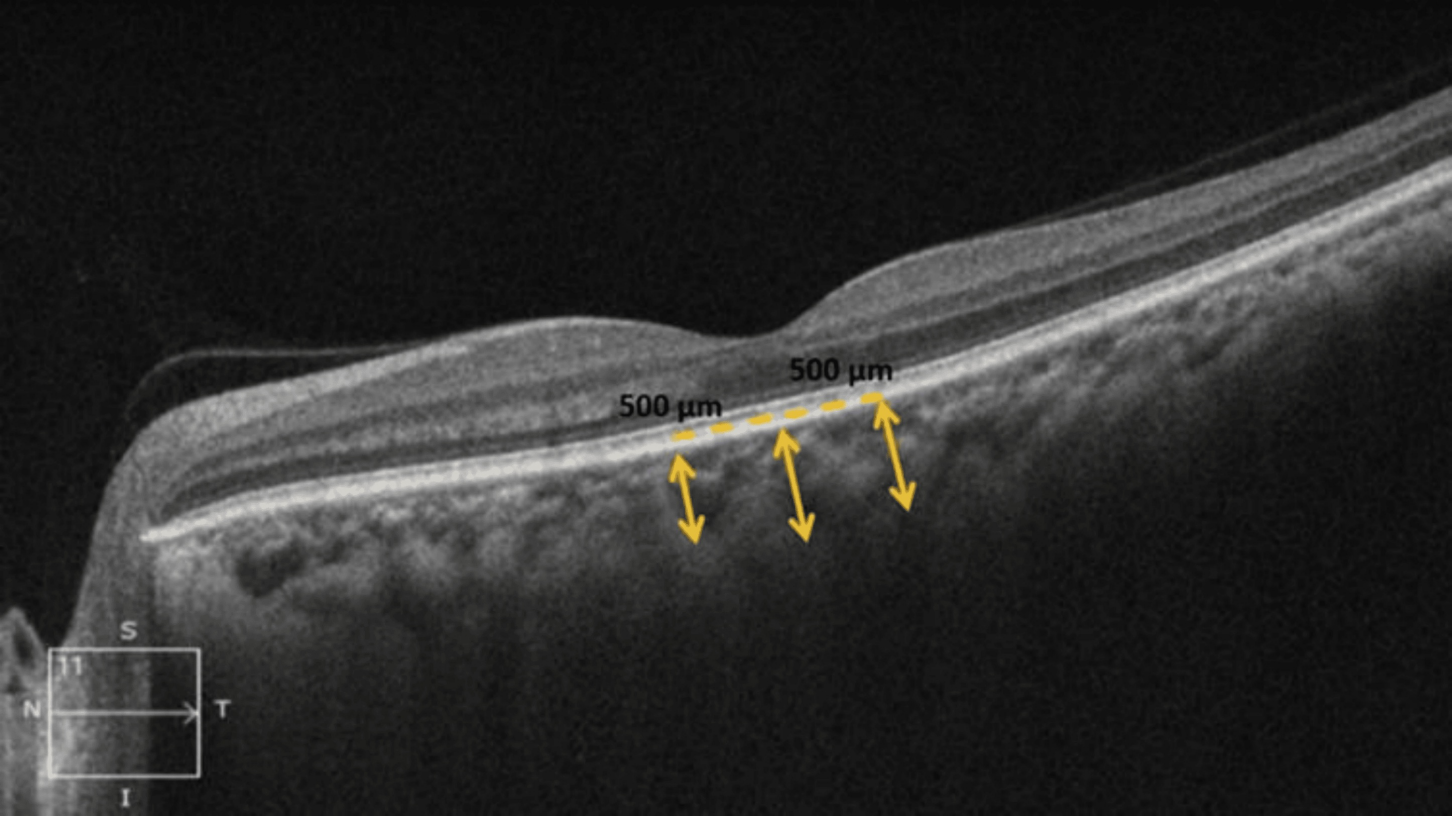
Representative SD-OCT image showing measurement of various choroidal thickness parameters of fellow eye in one of the RVO patients using EDI mode SD-OCT: spectral-domain optical coherence tomography; CT: choroidal thickness; RVO: retinal vascular occlusion; EDI: enhanced depth imaging

Figures 1-2 show the measurement of various CT parameters of the affected eye and fellow eye in one of our patients with SD-OCT scan using EDI mode, respectively.

Systemic confounders, such as hypertension and diabetes mellitus, were controlled by the paired comparison of affected and fellow eyes within individuals, though multivariate analysis was not performed due to the small sample size.

Fundus photography (FP) was done as per clinical requirements. Additional laboratory investigations were performed in selected patients as deemed necessary.

Data was tabulated in a Microsoft Excel spreadsheet (Microsoft Corporation, Redmond, USA) and was analyzed with the help of IBM SPSS Statistics for Windows, Version 28 (Released 2021; IBM Corp., Armonk, New York, USA). Presenting visual acuity in Snellen’s notation was converted into a LogMAR value for statistical analysis. Descriptive statistics for quantitative variables were summarized as mean ± standard deviation (SD). Percentages were reported for categorical variables. The inter-observer variability was calculated using an intra-class correlation coefficient test. Inferential statistics for comparison of quantitative variables between two groups included independent samples t-tests and paired samples t-tests. Correlation analysis was performed by Pearson’s correlation coefficient. A p-value less than 0.05 was considered statistically significant.

## Results

A total of 34 patients with unilateral RVO who fulfilled the inclusion and exclusion criteria during the study duration were considered for our study. The majority of the patients were males (n = 22; 64.7%). BRVO (n = 22; 64.7%) was found to be more common than CRVO (n = 12; 35.3%). Most of the patients (52.9%) did not have any associated comorbidities. The age-wise and gender-wise distribution and proportion of comorbidities in the study population have been summarized in Table 1.

**Table 1:** Age and gender distribution, types of vascular occlusion, and associated comorbidities SD: standard deviation; BRVO: branch retinal vein occlusion; CRVO: central retinal vein occlusion; RVO: retinal vascular occlusion

Parameter	Values
Age (in years) (mean ± SD (range))	
Mean age of study population	51.5 ± 7.6 (40-60)
Mean age of BRVO patients	50.7 ± 7.7 (40-60)
Mean age of CRVO patients	52.8 ± 7.5 (40-60)
Gender	
Male	22 (64.7%)
Female	12 (35.3%)
Type of RVO	
BRVO	22 (64.7%)
CRVO	12 (35.3%)
Associated comorbidities	
Hypertension	13 (38.2%)
Type 2 diabetes mellitus	3 (8.8%)
Nil	18 (52.9%)

There was a statistically significant difference in the presenting visual acuity between the affected and fellow eyes of RVO patients (p ≤ 0.001). Similarly, there was a statistically significant difference in the presenting visual acuity between the affected and fellow eyes in the BRVO and CRVO groups individually as well (p ≤ 0.001). No statistically significant difference was found between the AL and IOP between the affected and fellow eyes of RVO patients and also in the BRVO and CRVO groups. Table 2 shows the presenting visual acuity, IOP, and AL of the affected and fellow eyes of RVO patients.

**Table 2:** Presenting visual acuity, intraocular pressure and axial length of the affected and fellow eye of vascular occlusion patients * indicates paired samples t-test BRVO: branch retinal vein occlusion; CRVO: central retinal vein occlusion; IOP: intraocular pressure; SD: standard deviation

	Affected eye	Fellow eye	p-value*
Presenting visual acuity (in LogMAR) (mean ± SD (range))			
All vascular occlusion patients (n = 34)	0.7 ± 0.4 (0.0-1.3)	0.1 ± 0.2 (0.0-0.5)	<0.001
BRVO (n = 22)	0.6 ± 0.4 (0.0-1.3)	0.2 ± 0.2 (0.0-0.5)	<0.001
CRVO (n = 12)	0.7 ± 0.5 (0.0-1.3)	0.1 ± 0.2 (0.0-0.5)	<0.001
IOP (in mm of Hg) (mean ± SD (range))			
All vascular occlusion patients (n = 34)	16.2 ± 3.5 (11-24)	15.6 ± 3.8 (10-25)	0.12
BRVO (n = 22)	16.0 ± 3.3 (11-24)	15.1 ± 3.5 (10-23)	0.07
CRVO (n = 12)	16.7 ± 4.0 (12-24)	16.7 ± 4.1 (10-25)	1
Axial length (in mm) (mean ± SD (range))			
All vascular occlusion patients (n = 34)	22.7 ± 1.0 (20.0-24.0)	22.8 ± 1.1 (18.5-24.5)	0.34
BRVO (n = 22)	22.6 ± 1.0 (20.0-23.9)	22.6 ± 1.2 (18.5-24.0)	0.87
CRVO (n = 12)	23.1 ± 0.8 (21.7-24.0)	23.2 ± 0.9 (22.0-24.5)	0.06

The inter-observer variability between two readings by two different observers for different CT parameters (sub-foveal CT, 500 µm nasal, and 500 µm temporal CT) calculated using an intra-class correlation coefficient test showed the agreement between both readings for all CT parameters with an intra-class correlation coefficient of >0.9, suggesting a discordance of ≤10% between both observers.

The overall mean CT (mean of sub-foveal, 500 µm nasal, and 500 µm temporal regions) in affected eyes was significantly higher (p ≤ 0.001) than the mean CT in fellow eyes of RVO patients. Similarly, the independent sub-foveal, 500 µm nasal, and 500 µm temporal CT were also found to be significantly higher (p ≤ 0.001) in affected eyes as compared to fellow eyes of RVO patients.

While analyzing separately in BRVO as well as in CRVO subgroups, the overall mean CT in affected eyes was significantly higher than the mean CT in fellow eyes of BRVO patients (p ≤ 0.001), and the independent subfoveal, 500 µm nasal, and 500 µm temporal CT were also found to be significantly higher (p ≤ 0.001) in affected eyes as compared to fellow eyes of BRVO patients. A similar finding was noted for all thickness parameters in CRVO patients (p ≤ 0.05). Table 3 shows the summary of CT parameters.

**Table 3:** Summary of choroidal thickness parameters * indicates paired samples t-test SFCT: subfoveal choroidal thickness; CT: choroidal thickness; RVO: retinal vascular occlusion; BRVO: branch retinal vein occlusion; CRVO: central retinal vein occlusion; SD: standard deviation

	Affected eye	Fellow eye	p-value*
SFCT (in µm) (mean ± SD (range))			
All RVO (n = 34)	326.1 ± 80.0 (170.5-507.0)	264.2 ± 69.2 (136.0-427.0)	<0.001
BRVO (n = 22)	344.3 ± 78.5 (173.0-507.0)	271.3 ± 63.5 (136.0-410.0)	<0.001
CRVO (n= 12)	292.6 ± 74.3 (170.5-430.0)	251.2 ± 80.0 (138.0-427.0)	<0.001
Nasal 500 µ CT (in µm) (mean ± SD (range))			
All RVO (n = 34)	325.4 ± 99.7 (160.0-630.5)	263.1 ± 69.5 (107.0-416.0)	<0.001
BRVO (n = 22)	348.4 ± 104.9 (183.0-630.5)	273.4 ± 62.9 (156.5-416.0)	<0.001
CRVO (n = 12)	283.1 ± 76.3 (160.0-423.0)	244.2 ± 79.5 (107.0-396.5)	0.001
Temporal 500 µ CT (in µm) (mean ± SD (range))			
All RVO (n = 34)	328.1 ± 100.8 (150.5-660.5)	255.2 ± 72.6 (113.5-411.0)	<0.001
BRVO (n = 22)	348.1 ± 108.8 (170.0-660.5)	262.0 ± 68.6 (141.0-411.0)	<0.001
CRVO (n = 12)	291.4 ± 75.0 (150.5-431.5)	242.8 ± 80.9 (113.5-404.5)	0.003
Mean CT (in µm) (mean ± SD (range))			
All RVO (n = 34)	326.5 ± 87.8 (160.3-541.2)	260.8 ± 68.5 (119.5-412.3)	<0.001
BRVO (n = 22)	347.0 ± 89.7 (175.3-541.2)	268.9 ± 62.6 (144.5-412.3)	<0.001
CRVO (n = 12)	289.1 ± 73.3 (160.3-428.2)	246.1 ± 79.0 (119.5-409.3)	<0.001

There was no statistically significant correlation between mean CT, independent sub-foveal, 500 µm nasal, and 500 µm temporal CT and presenting visual acuity in affected eyes in RVO patients. Similarly, there was no statistically significant correlation between mean CT, independent sub-foveal, 500 µm nasal, and 500 µm temporal CT and presenting visual acuity in affected eyes in BRVO and CRVO patients separately. Table 4 shows the correlation analysis between various CT parameters and baseline visual acuity in affected eyes in RVO patients.

**Table 4:** Correlation analysis between various thickness parameters and baseline visual acuity in affected eyes in RVO patients * r indicates Pearson correlation; p indicates significant value of Pearson correlation test CT: choroidal thickness; SD: standard deviation; SFCT: subfoveal choroidal thickness; BRVO: branch retinal vein occlusion; CRVO: central retinal vein occlusion

	Thickness parameter in the affected eye (in µm) (mean ± SD (range))	Presenting visual acuity in the affected eye (in LogMAR) (mean ± SD (range))	Statistical significance*
Mean CT			
Overall (n = 34)	326.5 ± 87.8 (160.3-541.2)	0.7 ± 0.4 (0.0-1.3)	r = -0.15; p = 0.40
BRVO (n = 22)	347.0 ± 89.7 (175.3-541.2)	0.6 ± 0.4 (0.0-1.3)	r = -0.04; p = 0.88
CRVO (n = 12)	289.0 ± 73.3 (160.3-428.2)	0.7 ± 0.5 (0.0-1.3)	r = -0.35; p = 0.27
SFCT			
Overall (n = 34)	326.1 ± 80.0 (170.5-507.0)	0.7 ± 0.4 (0.0-1.3)	r = -0.34; p = 0.05
BRVO (n = 22)	344.3 ± 78.5 (173.0-507.0)	0.6 ± 0.4 (0.0-1.3)	r = -0.32; p = 0.15
CRVO (n = 12)	292.6 ± 74.3 (170.5-430.0)	0.7 ± 0.5 (0.0-1.3)	r = -0.36; p = 0.26
Nasal 500 µm CT			
Overall (n = 34)	325.4 ± 99.7 (160.0-630.5)	0.7 ± 0.4 (0.0-1.3)	r = -0.07; p = 0.72
BRVO (n = 22)	348.4 ± 104.9 (183.0-630.5)	0.6 ± 0.4 (0.0-1.3)	r = 0.03; p = 0.89
CRVO (n = 12)	283.1 ± 76.3 (160.0-423.0)	0.7 ± 0.5 (0.0-1.3)	r = -0.24; p = 0.46
Temporal 500 µm CT			
Overall (n = 34)	328.1 ± 100.8 (150.5-660.5)	0.7 ± 0.4 (0.0-1.3)	r = -0.05; p = 0.76
BRVO (n = 22)	348.1 ± 108.8 (170.0-660.5)	0.6 ± 0.4 (0.0-1.3)	r = 0.11; p = 0.62
CRVO (n = 12)	291.4 ± 75.0 (150.5-431.5)	0.7 ± 0.5 (0.0-1.3)	r = -0.43; p = 0.17

## Discussion

In our study, the mean age of presentation for RVO disorders was 51.47 ± 7.60 years in the select range of 40 to 60 years to eliminate the effect of age on CT. Males (64.7%) were affected nearly 1.8 times more than females (35.3%). This could be because males in India have easier access to medical care than females. Large population-based studies like the Beaver Dam Dye Study, the Blue Mountains Eye Study, and the Beijing Eye Study have also reported male preponderance [3,17-18].

Newman-Casey et al., in their study on risk factors associated with developing BRVO, showed that 8.6% of the study population had hypertension and 0.6% of the study population had type 2 diabetes mellitus [18]. In our study, 38.2% of the 34 patients had hypertension, 8.8% had type 2 diabetes mellitus, and 52.9% had no co-morbidities.

Zhou et al. studied age-related changes in CT in 144 participants aged between 20 and 88 years. They found out that the CT decreases with age (p < 0.0001) [19]. Flores-Moreno et al. in their study found that CT becomes thinner with an increase in the AL of the eye (p ≤ 0.001) [13]. Hata et al., in a study, showed that choroidal thinning was associated with an increase in IOP [20].

However, few studies do not mention the baseline AL and IOP of the RVO-affected eyes, which might have an impact on the CT of the eye [21-23]. We measured the AL and IOP of all the RVO-affected eyes and the fellow eyes in our study. No statistically significant difference between the AL of RVO-affected and fellow eyes (p = 0.335) as well as between the IOP of the RVO-affected and fellow eyes (p = 0.120) was found in our study. Thus, we eliminated the chances of AL and IOP affecting the CT of the eyes in our study.

Only ICGA, laser Doppler flowmetry, and ultrasound could be used to examine the choroid until recently. Although the above techniques can detect choroidal vascular abnormalities and changes in blood flow, SD-OCT helps to obtain three-dimensional anatomic information regarding the RPE and choroid layers. OCT is a non-invasive imaging technique for obtaining high-resolution retinal cross-sectional scans. Spaide has published a new acquisition technique employing the Spectralis OCT equipment called EDI. Placing the obtained structures closer to zero delay allows a better view of the choroid [24].

A number of recent studies have looked at CT in RVO patients [3,15,22,24-30]. Kim et al. measured regional CT in 57 BRVO eyes at baseline. The mean CT of the occlusive area in BRVO eyes was significantly higher than that of the non-occlusive, sub-foveal, and corresponding areas in the fellow eyes [7]. Tsuiki et al. measured the mean SFCT in 36 CRVO patients. Compared with the fellow eyes, the CRVO eyes showed significantly greater SFCT. In this study, the mean SFCT was 257.1 ± 83.2 µm in the CRVO eyes and 222.6 ± 67.8 µm in the fellow eyes [29]. The Beijing Eye Study, on the other hand, found that CT in RVO eyes was comparable to the normal contralateral eyes [3]. In these studies, patients did not have recent-onset RVO, and there was no significant ME on examination or OCT imaging. Akin to the above-mentioned studies, our study also demonstrated that the CT of RVO-affected eyes is significantly greater than that of the fellow eyes.

In our study, the mean CT, SFCT, CT 500 µm nasal, and 500 µm temporal to the fovea in the RVO-affected eyes were found to be higher than the fellow eyes. The difference between the average sub-foveal, 500 µm nasal, 500 µm temporal CT, and the average CT of the RVO-affected and fellow eyes was found to be statistically significant (p ≤ 0.001).

Because the choroid has been discovered to be extremely responsive to VEGF, the fact that baseline CT is enhanced in RVO is not surprising. The hypothesis provided by Rayess et al. is that increased intraocular VEGF levels have been seen in RVO patients due to retinal ischemia, which is thought to cause increased choroidal vascular permeability and, as a result, an increase in CT. The increased baseline CT in CRVO may reflect the elevated VEGF levels and help predict which eyes may be most responsive to anti-VEGF therapy. Because the choroid is involved in maintaining perfusion to the outer retinal layers and is the sole source of metabolic exchange for the fovea, a thicker choroid may reflect better-preserved perfusion to the outer retina during RVO, allowing for greater visual gains after anti-VEGF therapy reduces edema [30].

Direct fluid transfer from blocked arteries to nearby tissues can explain regional differences in CT in the current investigation. Damaged vascular endothelial cells, vessel dilatation, and increased ocular blood flow in the occluded retinal arteries might cause localized extravascular fluid leakage and/or increased hydrostatic pressure.

In our cross-sectional study, where participants were recruited prospectively, we assessed the relationship between presenting visual acuity and baseline thickness parameters in the affected eyes of treatment-naive RVO patients. Visual acuity was compared with mean CT, sub-foveal CT, 500 µm nasal CT, 500 µm temporal CT, and CRT. A statistically significant positive correlation was found only between baseline CRT and presenting visual acuity (p = 0.036), indicating that ME is a key determinant of visual impairment. No significant correlations were observed between mean CT (p = 0.404), sub-foveal CT, 500 µm nasal CT, or 500 µm temporal CT and visual acuity in the overall RVO cohort, nor in the BRVO (p = 0.88) or CRVO (p = 0.27) subgroups separately, as shown in Table 4, suggesting that CT does not directly influence baseline visual function in early RVO.

The prospective inclusion of participants using a comparative approach is one of our study's noteworthy strengths. We included patients of the age group 40 to 60 years only to eliminate the effect of age on CT. We considered the baseline AL and IOP of the affected and fellow eyes so that these parameters do not have any effect on the CT. All the CT parameters were measured by two independent, experienced observers, and an average of the readings was considered for statistical analysis. We evaluated each eye separately and took bilateral CT measurements at several topographical locations, such as 500 µm nasal, 500 µm temporal to fovea, and sub-foveal. Unlike the majority of other research that only measured unilateral sub-foveal CT, we were able to make more accurate conclusions on extensive choroidal changes by validating our data in both eyes at different choroidal areas. We also tried to find out whether there is any correlation between the presenting visual acuity and the baseline CT in RVO patients.

There are also some limitations in our study. The primary limitations of the current investigation include the absence of swept-source OCT, manual in-built caliper-based CT measurement, and the unavailability of longitudinal follow-up of RVO patients. The results of our study might not apply to other ethnic groups because it was carried out in a comparatively smaller homogeneous community with similar demographic backgrounds. Our study consisted of a small sample size, which was a cohort of both BRVO and CRVO cases. The number of CRVO cases (n = 12) was less as compared to the number of BRVO cases (n = 22). Our study also had a limitation in that further subgroup analysis was not performed.

## Conclusions

In conclusion, we found a statistically significant difference between the average sub-foveal, 500 µm nasal, and 500 µm temporal CT of the RVO-affected eyes and the fellow eyes. There was no statistical significance between the correlation of visual acuity and various CT parameters of affected eyes of RVO patients. Future prospective studies involving larger sample sizes and multiple longitudinal follow-ups may further enlighten the possible role of CT as a biomarker in RVO patients.

## References

[1] Hayreh SS (2005). Prevalent misconceptions about acute retinal vascular occlusive disorders. Prog Retin Eye Res.

[2] Hayreh SS, Zimmerman B, McCarthy M, Podhajsky P (2001). Systemic diseases associated with various types of retinal vein occlusion. Am J Ophthalmol.

[3] Hayreh SS, Zimmerman MB, Podhajsky P (1994). Incidence of various types of retinal vein occlusion and their recurrence and demographic characteristics. Am J Ophthalmol.

[4] Pieramici DJ, Rabena M, Castellarin AA (2008). Ranibizumab for the treatment of macular edema associated with perfused central retinal vein occlusions. Ophthalmology.

[5] Shalchi Z, Mahroo O, Bunce C, Mitry D (2020). Anti-vascular endothelial growth factor for macular oedema secondary to branch retinal vein occlusion. Cochrane Database Syst Rev.

[6] Du KF, Xu L, Shao L (2013). Subfoveal choroidal thickness in retinal vein occlusion. Ophthalmology.

[7] Kim KH, Lee DH, Lee JJ, Park SW, Byon IS, Lee JE (2015). Regional choroidal thickness changes in branch retinal vein occlusion with macular edema. Ophthalmologica.

[8] Marisse M, Erick H, Lihteh W (2011). Choroidal thickness in patients with systemic hypertension. Invest Ophthalmol Vis Sci.

[9] Tian J, Marziliano P, Baskaran M, Tun TA, Aung T (2012). Automatic measurements of choroidal thickness in EDI-OCT images. Conf Proc IEEE Eng Med Biol Soc.

[10] Waghamare SR, Mittal S, Pathania M, Samanta R, Kumawat D, Gupta N, Mittal SK (2021). Comparison of choroidal thickness in systemic hypertensive subjects with healthy individuals by spectral domain optical coherence tomography. Indian J Ophthalmol.

[11] Naharwal A, Samanta R, Agrawal A, Chawla L, Gaurav A, Jayaraj S (2025). Comparison of subfoveal choroidal thickness in pre-eclamptic, healthy pregnant, and non-pregnant women. Indian J Ophthalmol.

[12] Borooah S, Sim PY, Phatak S (2021). Pachychoroid spectrum disease. Acta Ophthalmol.

[13] Flores-Moreno I, Lugo F, Duker JS, Ruiz-Moreno JM (2013). The relationship between axial length and choroidal thickness in eyes with high myopia. Am J Ophthalmol.

[14] Jonas JB, Forster TM, Steinmetz P, Schlichtenbrede FC, Harder BC (2014). Choroidal thickness in age-related macular degeneration. Retina.

[15] Klein R, Klein BE, Moss SE, Meuer SM (2000). The epidemiology of retinal vein occlusion: the Beaver Dam Eye Study. Trans Am Ophthalmol Soc.

[16] Mitchell P, Smith W, Chang A (1996). Prevalence and associations of retinal vein occlusion in Australia: the Blue Mountains Eye Study. Arch Ophthalmol.

[17] Zhou JQ, Xu L, Wang S (2013). The 10-year incidence and risk factors of retinal vein occlusion: the Beijing Eye Study. Ophthalmology.

[18] Newman-Casey PA, Stem M, Talwar N, Musch DC, Besirli CG, Stein JD (2014). Risk factors associated with developing branch retinal vein occlusion among enrollees in a United States managed care plan. Ophthalmology.

[19] Zhou H, Dai Y, Shi Y (2020). Age-related changes in choroidal thickness and the volume of vessels and stroma using swept-source OCT and fully automated algorithms. Ophthalmol Retina.

[20] Hata M, Hirose F, Oishi A, Hirami Y, Kurimoto Y (2012). Changes in choroidal thickness and optical axial length accompanying intraocular pressure increase. Jpn J Ophthalmol.

[21] Spaide RF, Koizumi H, Pozzoni MC (2008). Enhanced depth imaging spectral-domain optical coherence tomography. Am J Ophthalmol.

[22] Gutman F, Zegarra H (1984). Macular edema secondary to occulusion of the retinal veins. Surv Ophthalmol.

[23] Hayreh SS, Zimmerman MB (2014). Branch retinal vein occlusion: natural history of visual outcome. JAMA Ophthalmol.

[24] Adhi M, Duker JS (2013). Optical coherence tomography - current and future applications. Curr Opin Ophthalmol.

[25] Ruiz-Medrano J, Flores-Moreno I, Peña-García P, Montero JA, Duker JS, Ruiz-Moreno JM (2014). Macular choroidal thickness profile in a healthy population measured by swept-source optical coherence tomography. Invest Ophthalmol Vis Sci.

[26] Tan CS, Ouyang Y, Ruiz H, Sadda SR (2012). Diurnal variation of choroidal thickness in normal, healthy subjects measured by spectral domain optical coherence tomography. Invest Ophthalmol Vis Sci.

[27] Manjunath V, Taha M, Fujimoto JG, Duker JS (2010). Choroidal thickness in normal eyes measured using Cirrus HD optical coherence tomography. Am J Ophthalmol.

[28] Kang HM, Choi JH, Koh HJ, Lee CS, Lee SC (2018). Significant reduction of peripapillary choroidal thickness in patients with unilateral branch retinal vein occlusion. Retina.

[29] Tsuiki E, Suzuma K, Ueki R, Maekawa Y, Kitaoka T (2013). Enhanced depth imaging optical coherence tomography of the choroid in central retinal vein occlusion. Am J Ophthalmol.

[30] Rayess N, Rahimy E, Ying GS (2016). Baseline choroidal thickness as a predictor for treatment outcomes in central retinal vein occlusion. Am J Ophthalmol.

